# Gut microbiota and metabolites in lipid metabolism and intramuscular fat deposition: mechanisms and implications for meat quality

**DOI:** 10.1186/s40104-025-01279-6

**Published:** 2025-11-13

**Authors:** Xiaofeng Song, Chenglong Jin, Ruifan Wu, Yongjie Wang, Xiaofan Wang

**Affiliations:** 1 College of Animal Science, State Key Laboratory of Swine and Poultry Breeding Industry/National Engineering Research Center for Breeding Swine Industry/Guangdong Provincial Key Laboratory of Animal Nutrition Control, South China Agricultural University, Guangzhou, 510642 China; 2 Institute of Animal Science, Guangdong Academy of Agricultural Sciences/Key Laboratory of Animal Nutrition and Feed Science in South China, Ministry of Agriculture and Rural Affairs/Guangdong Provincial Key Laboratory of Animal Breeding and Nutrition, Guangzhou, 510640 China; 3 Department of Animal Sciences, College of Agriculture and Environmental Sciences, North Carolina Agricultural and Technical State University, Greensboro, NC 27411 USA

**Keywords:** Bile acids, Branched-chain amino acids, Gut microbiota, Intramuscular fat deposition, Short-chain fatty acids

## Abstract

Intramuscular fat (IMF) content serves as the key determinants of meat quality. Emerging evidence indicates that gut microbiota and their metabolites significantly influence IMF deposition levels by modulating host lipid metabolism through multiple pathways, positioning microbial regulation as a pivotal target for meat quality improvement. However, existing studies remain fragmented, predominantly focusing on isolated mechanisms or correlations without a systematic view of the regulatory network. This review consolidates the core mechanisms through which microbiota-derived metabolites including short-chain fatty acids, bile acids, branched-chain amino acids, trimethylamine N-oxide, tryptophan derivatives, succinate, polyamines etc., regulate IMF deposition and proposes a targeted intervention framework, the “gut microbiota/metabolites-IMF axis”. By integrating these insights, we provide a theoretical foundation and define practical research pathways to assess the potential of microbial-based strategies for improving meat quality in swine production.

## Introduction

Pork ranks among the most significant and extensively produced meats globally. According to the Food and Agriculture Organization of the United Nations, global pork production reached 123 million tons in 2022, accounting for 34% of total meat production worldwide [1]. In recent decades, pig husbandry has focused on achieving higher lean meat rates and reducing backfat thickness. However, enhancing the quality of pork has increasingly become a central topic of research [2, 3]. Indicators such as meat color, tenderness, pH, flavor, marbling score, and juiciness are key to evaluating pork quality [4]. Intramuscular fat (IMF) plays a pivotal role, significantly influencing tenderness, marbling, and flavor, thereby serving as a critical determinant of meat quality. A moderate and evenly distributed amount of marbling significantly enhances the flavor, juiciness, and overall palatability of meat, with an ideal IMF content of 2.5% to 3.5% in pork optimally improving consumer acceptability [5]. However, excessive or uneven marbling may raise health concerns related to fat intake and increase spoilage risks during processing, while insufficient IMF leads to dry, tasteless meat and higher processing spoilage susceptibility [6]. Thus, achieving balanced marbling is crucial for both quality and human health.

Triglycerides, cholesterol, and phospholipids constitute the lipids in pork and their accumulation is predominantly influenced by factors such as age, sex, breed, nutritional level, and genetics [7, 8]. Importantly, growing evidence indicates that the gut microbiota can regulate host lipogenesis through multifaceted mechanisms [9, 10]. The gut microbiome dynamically modulates host homeostasis like metabolism and energy balance, which affects lipid storage and utilization in skeletal muscle [11]. Additionally, gut microbiota can produce various metabolites that influence host lipid metabolism, including short-chain fatty acids (SCFAs), bile acids (BAs), branched-chain amino acids (BCAAs), trimethylamine N-oxide (TMAO), tryptophan derivatives, and other microbial-derived products like succinate or polyamines [12–17]. However, research on the "gut microbiota-derived metabolites-IMF axis" remains relatively fragmented, primarily due to spatiotemporal separation, methodological limitations, and inherent systemic complexity.

These challenges have hindered a comprehensive elucidation of the molecular mechanisms underlying bacterial-intramuscular fat interactions. To address this gap, we conducted a comprehensive and systematic review of recent literature on gut microbiome-mediated regulation of lipid metabolism. We first demonstrated how gut microbiota and their metabolites regulate lipid redistribution and muscle energy metabolism. Then, we identified key targets and signaling pathways in microbe-host interactions, proposed microbiota-based strategies for meat quality improvement, and discussed current research gaps with future directions for multi-omics approaches.

## Cellular basis and molecular regulation of IMF deposition

IMF is the lipid found within and between muscle bundles and fibers, accumulating progressively with the development of connective tissue. The accumulation of IMF primarily involves an increase in the number of intramuscular adipocytes and the buildup of lipid droplets within these adipocytes and between muscle fibers. The proliferation and hypertrophy of intramuscular adipocytes are the primary mechanisms contributing to IMF deposition [18]. The increase in adipocytes primarily occurs during the embryonic stage and early phases of growth and development. During the embryonic period, mesenchymal pluripotent stem cells in the mesoderm gradually differentiate into adipocytes [19].

Intramuscular adipocytes originate from various sources of stem cells or progenitor cells, including mesenchymal stem cells (MSCs), pericytes, and side population cells. Among these, a subset of MSCs known as fibro/adipogenic progenitors has been reported to be the primary precursor cells for IMF [20, 21]. This cell subpopulation, marked by platelet-derived growth factor receptor α, exhibits bidirectional potential for both fibrogenic and adipogenic differentiation and can be directed to differentiate into adipogenic precursor cells under the regulation of specific signals [22]. Lipogenesis is governed by a sophisticated transcriptional network in which peroxisome proliferator-activated receptor γ (PPARγ) and CCAAT/enhancer-binding proteins (C/EBPs) function as master regulators, orchestrating adipogenesis, de novo lipogenesis, lipid storage, and systemic insulin sensitivity through interorgan crosstalk among liver, muscle, and adipose tissues [23, 24]. During terminal differentiation, mature white adipocytes utilize cell death-inducing DNA fragmentation factor 45-like effector (CIDE) family proteins (particularly CIDEC) to mediate the fusion of small lipid droplets, ultimately forming a unilocular central lipid droplet that occupies over 90% of the cytoplasmic volume. This process is accompanied by the coating of the lipid droplet surface with perilipin family proteins (predominantly PLIN1), which maintains structural stability and regulates lipolysis [25].

**Fig. 1:**
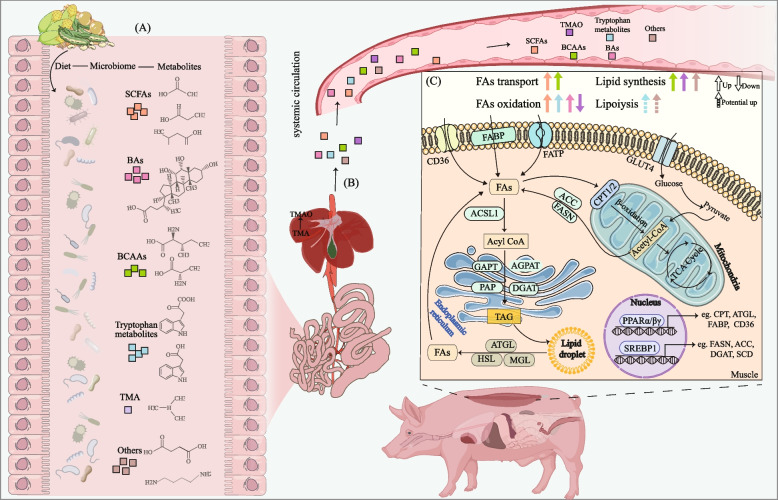
Roles of gut microbiota-derived metabolites on intramuscular fat deposition of pig. **A** Gut microbiota metabolizes dietary nutrients to generate diverse metabolites, including SCFAs, BAs, BCAAs, tryptophan metabolites, TMA and others. These metabolites cross the intestinal barrier, enter systemic circulation via the portal vein, and reach the liver, undergoing further processing (e.g., hepatic conversion of TMA to trimethylamine N-oxide, TMAO). **B** Subsequently, these microbial metabolites enter systemic circulation via the hepatic vein, traveling through the bloodstream to muscle tissue. **C** Within the muscle, these compounds modulate lipid metabolism by regulating fatty acid transport, β-oxidation, lipid synthesis, and lipolysis. Imported fatty acids undergo either esterification into TAG for storage as lipid droplets or mitochondrial import to fuel energy production. The intracellular lipid droplets’ catabolism provides an additional endogenous source of fatty acids. Acetyl-CoA is a critical metabolic intermediate and an essential substrate for de novo fatty acid synthesis. In muscle tissue, imported glucose can be converted to pyruvate and metabolized to generate this pivotal intermediate. *SCFAs* Short-chain fatty acids, *BAs* Bile acids, *BCAAs* Branched-chain amino acids, *TMA* Trimethylamine, *TMAO* Trimethylamine N-oxide, *FAs* Fatty acids, *TAG* Triglyceride, *PPARγ* Peroxisome proliferator-activated receptor gamma, *C/EBPs* CCAAT/enhancer-binding proteins, *SREBP1* Sterol regulatory element-binding protein 1, *FABP* Fatty acid-binding protein, *CD36* Cluster of differentiation 36, *FATP1* Fatty acid transport protein 1, *GLUT4* Glucose transporter type 4, *ACSL1* Acyl-CoA synthetase long-chain family member1, *GAPT* Glycerol-3-phosphate acyltransferase, *AGPAT* 1-acylglycerol-3-phosphate O-acyltransferase, *PAP* Phosphatidic acid phosphatase, *DGAT* Diacylglycerol O-acyltransferase 2, *FASN* Fatty acid synthase, *ACC* Acetyl-CoA carboxylase, *ACACA* Acetyl-CoA carboxylase alpha, *CPT1* Carnitine palmitoyltransferase1, *ATGL* Adipose triglyceride lipase, *HSL* Hormone-sensitive lipase, *MGL* Monoglyceride lipase, *TCA cycle* Tricarboxylic acid cycle, *SCD* Stearoyl-CoA desaturase

The dynamic equilibrium of lipid droplet deposition depends on the precise regulation of fatty acid uptake and oxidation. In muscle tissue, fatty acids undergo metabolic partitioning: a portion is directed toward mitochondrial β-oxidation for energy production in myofibers, while another portion is selectively taken up by intramuscular adipocytes and channeled into the triacylglycerol (TAG) biosynthetic pathway to promote IMF deposition (Fig. 1) [26, 27]. In skeletal muscle, TAG biosynthesis primarily relies on the glycerol-3-phosphate acylation pathway. This process involves the sequential esterification of three fatty acid molecules onto a glycerol backbone, forming this crucial energy storage molecule [28]. Regarding fatty acid sources, animals can generate palmitic acid either through de novo synthesis mediated by acetyl-CoA carboxylase (ACC) and fatty acid synthase (FASN), or by acquiring exogenous fatty acids from dietary sources. These exogenous fatty acids are subsequently taken up into muscle tissue via fatty acid transporters such as cluster of differentiation 36 (CD36) and fatty acid-binding protein 4 (FABP4) [8]. The fate of fatty acids is governed by key metabolic nodes. Under energy-sufficient conditions, elevated levels of malonyl-CoA (the product of ACC) allosterically inhibit carnitine palmitoyltransferase 1 (CPT1) on the mitochondrial membrane, thereby blocking long-chain fatty acid entry into mitochondria for oxidation and diverting them toward TAG synthesis. Conversely, during fasting or energy stress, AMP-activated protein kinase (AMPK)-mediated phosphorylation inhibits ACC, markedly reducing malonyl-CoA levels and relieving CPT1 inhibition [29–31]. This dynamic regulatory mechanism precisely balances cellular energy storage (lipid droplet deposition) and mobilization (β-oxidation), directly influencing IMF deposition efficiency and meat quality traits. We have summarized the major genes influencing IMF identified in transcriptomic studies in recent years (Table 1). IMF content is a complex trait regulated by multiple factors including animal genetics, age, breed, nutrition, and gut microbiota [13, 44–48]. Recent reports also identify the gut microbiota as one of the primary influences on IMF deposition in animals.

**Table 1:** Genes related to intramuscular fat identified in transcriptomic

Gene	Signaling pathway	Lipid metabolism	Methods	Citation
*CD36*	AMPK signaling pathway	FA transport and uptake	Multi-omics	[32]
*FABP4*	PPAR signaling pathway	FA transport and uptake	Multi-omics; Molecular biology techniques	[32, 33]
*FABP5*	PPAR signaling pathway	FA transport and uptake	Multi-omics; Molecular biology techniques	[33]
*ACSL1*	PPAR, AMPK signaling pathway	FA beta oxidation; Lipid synthesis	Correlation analysis	[34]
*ADIPOR1*	AMPK signaling pathway	FA beta oxidation	Multi-omics; Molecular biology techniques	[35]
*ADIPOR2*	AMPK signaling pathway	FA beta oxidation	Multi-omics; Molecular biology techniques	[35]
*BDH1*	—	FA beta oxidation	Correlation analysis	[36]
*CPT1*	PPAR, AMPK signaling pathway	FA beta oxidation	Multi-omics; Molecular biology techniques	[34, 35]
*CPT2*	PPAR, AMPK signaling pathway	FA beta oxidation	Multi-omics; Molecular biology techniques	[35]
*CYP4B1*	—	FA beta oxidation	Multi-omics	[37]
*NR4A1*	—	FA beta oxidation	Correlation analysis; Molecular biology techniques	[38]
*PGC-1α*	AMPK signaling pathway	FA beta oxidation	Multi-omics; Molecular biology techniques	[35]
*PRKAG2*	AMPK signaling pathway	FA beta oxidation	Correlation analysis	[36]
*PPARA*	PPAR signaling pathway	FA beta oxidation	Correlation analysis	[36]
*PRKAA1*	AMPK signaling pathway	FA beta oxidation	Correlation analysis; Molecular biology techniques	[38]
*ADIPOQ*	PPAR, AMPK signaling pathway	Adipogenesis	Correlation analysis; Multi-omics	[35, 37, 39]
*APC*	Wnt signaling pathway	Adipogenesis	Multi-omics	[40]
*CEBP*	—	Adipogenesis	Multi-omics	[27, 41]
*CIDEC*	PPAR signaling pathway	Adipogenesis	Multi-omics	[37]
*CREB5*	AMPK signaling pathway	Adipogenesis	Multi-omics	[40]
*IRS1*	Insulin signaling pathway	Adipogenesis	Correlation analysis	[34]
*IRS2*	Insulin signaling pathway	Adipogenesis	Correlation analysis	[34, 36]
*LEP*	AMPK, JAK-STAT signaling pathway	Adipogenesis	Correlation analysis; Multi-omics	[35–37, 39]
*PCK1*	PI3K-Akt, AMPK, Insulin signaling pathway	Adipogenesis	Correlation analysis; Multi-omics	[35, 39]
*PPARG*	PPAR, AMPK signaling pathway	Adipogenesis	Correlation analysis; Multi-omics	[27, 35, 39]
*SREBF1*	—	Adipogenesis	Multi-omics	[41]
*ACACA*	—	Lipid synthesis	Correlation analysis; Molecular biology techniques	[38]
*ACC1*	AMPK signaling pathway	Lipid synthesis	Correlation analysis; Multi-omics; Molecular biology techniques	[35, 39, 42]
*DGAT2*	—	Lipid synthesis	Multi-omics; Correlation analysis; Molecular biology techniques	[37, 38]
*ELOVL1*	—	Lipid synthesis	Multi-omics; Molecular biology techniques	[33]
*ELOVL6*	—	Lipid synthesis	Correlation analysis; Molecular biology techniques	[33, 38]
*FASN*	AMPK signaling pathway	Lipid synthesis	Correlation analysis; Multi-omics; Molecular biology techniques	[35, 39, 42]
*GK*	PPAR signaling pathway	Lipid synthesis	Correlation analysis	[36]
*PIK3C*	PI3K/Akt signaling pathway	Lipid synthesis; Lipolysis	Correlation analysis	[34]
*SCD*	PPAR, AMPK signaling pathway	Lipid synthesis	Correlation analysis; Multi-omics; Molecular biology techniques	[35, 37, 39]
*THRSP*	—	Lipid synthesis	Multi-omics	[37]
*LIPE*	AMPK signaling pathway	Lipolysis	Multi-omics; Molecular biology techniques	[33, 35]
*PLIN1*	—	Lipolysis	Multi-omics; Correlation analysis; Molecular biology techniques	[34, 35, 37]
*PLIN2*	—	Lipolysis	Correlation analysis	[34]
*LPL*	—	Lipolysis	Multi-omics; Correlation analysis; Molecular biology techniques	[33, 38, 43]

*CD36* Cluster of differentiation 36, *FABP4* Fatty acid-binding protein 4, *FABP5* Fatty acid-binding protein 5, *ACSL1* Acyl-CoA synthetase long-chain family member 1, *ADIPOR1* Adiponectin receptor 1, ADIPOR2 Adiponectin receptor 2, *BDH1* 3-hydroxybutyrate dehydrogenase 1, *CPT1* Carnitine palmitoyltransferase 1, *CPT2* Carnitine palmitoyltransferase 2, *CYP4B1* Cytochrome P450 family 4 subfamily B member 1, *NR4A1* Nuclear receptor subfamily 4 group A member 1, *PGC-1α* Peroxisome proliferator-activated receptor gamma coactivator 1-alpha, *PRKAG2* Protein kinase AMP-activated non-catalytic subunit gamma 2, *PPARA* Peroxisome proliferator-activated receptor alpha, *PRKAA1* Protein kinase AMP-activated catalytic subunit alpha 1, *ADIPOQ* Adiponectin, *CEBPA* CCAAT/enhancer-binding protein alpha, *CIDEC* Cell death-inducing DFFA-like effector c, *IRS1* Insulin receptor substrate 1, *IRS2* Insulin receptor substrate 2, *LEP* Leptin, *PCK1* Phosphoenolpyruvate carboxykinase 1, *PPARG* Peroxisome proliferator-activated receptor gamma, *SREBF1* Sterol regulatory element-binding transcription factor 1, *ACACA* Acetyl-CoA carboxylase alpha, *ACC1* Acetyl-CoA carboxylase 1, *DGAT2* Diacylglycerol O-acyltransferase 2, *ELOVL* ELOVL fatty acid elongase, *FASN* Fatty acid synthase, *GK* Glycerol kinase, *PIK3CA* Phosphatidylinositol-4,5-bisphosphate 3-kinase catalytic subunit alpha, *PIK3CB* Phosphatidylinositol-4,5-bisphosphate 3-kinase catalytic subunit beta, *SCD* Stearoyl-CoA desaturase, *THRSP* Thyroid hormone responsive protein, *LIPE* Lipase E, hormone-sensitive type, *PLIN1* Perilipin 1, *PLIN2* Perilipin 2, *LPL* Lipoprotein lipase, AMPK AMP-activated protein kinase, *PPAR* Peroxisome proliferator-activated receptor, *JAK-STAT* Janus kinase-signal transducer and activator of transcription, *PI3K-Akt* Hosphoinositide 3-kinase-v-akt murine thymoma viral oncogene homolog

## Gut microbiota metabolites and lipid metabolism

Within the regulatory network of lipid metabolism, gut microbiota exerts significant influence through their metabolic outputs. A growing body of evidence demonstrates that microbial-derived metabolites directly modulate lipid metabolic pathways (Fig. 1), establishing this axis as an active area of investigation.

### Short-chain fatty acids

**Fig. 2:**
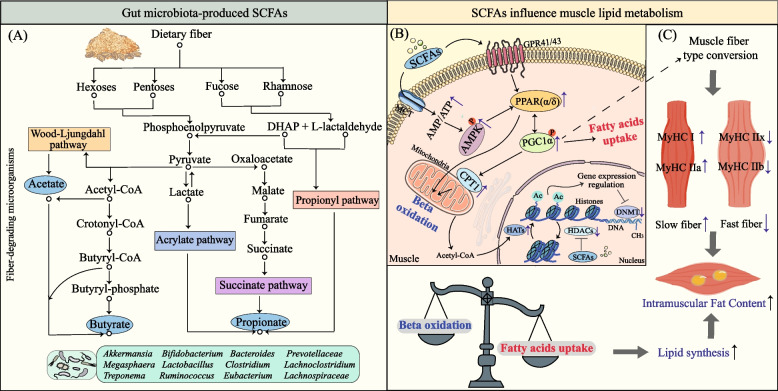
Gut microbiota-derived SCFAs on host intramuscular lipid metabolism. **A** Microbial fermentation of dietary fibers to produce SCFAs, such as acetate, butyrate, and propionate. In this process, succinate serves as an intermediate metabolite in microbial SCFA synthesis. Key microbial species involved in dietary fiber fermentation are listed at the bottom. **B** The SCFAs regulate muscle lipid metabolism. SCFAs, mainly acetate, propionate, and butyrate, are transported into muscle cells via monocarboxylate transporters (MCTs) or promote fatty acid uptake and oxidation by activating AMPK and PPAR signaling pathways through GPR41/43 receptors. **C** SCFAs may induce a shift toward slow-twitch muscle fibers and modulate gene expression via epigenetic modifications. Notably, intramuscular fat accumulation increases markedly when fatty acid uptake significantly exceeds oxidation. *DHAP* Dihydroxyacetone phosphate, *MCTs* Monocarboxylate transporters, *GPR41/43* G protein-coupled receptor 41/43, *AMPK* AMP-activated protein kinase, *PGC-1α* Peroxisome proliferator-activated receptor gamma coactivator 1-alpha, *HATs* Histone acetyltransferases, *HDACs* Histone deacetylases, *DNMT* DNA methyltransferase, *MyHC* Myosin heavy chain. Other abbreviations as in Fig. 1

The gut microbiota including *Bacteroidetes*, *Bifidobacterium*, *Prevotella, Ruminococcus*, *Treponema*, *Clostridium butyricum*, assists the host in digesting resistant carbohydrates (such as cellulose, resistant starch, β-glucans, and inulin) through carbohydrate-active enzymes, which generate substantial amounts of SCFAs in the intestine [49–51]. The abundance of these specific bacterial genera involved in polysaccharide degradation significantly correlates with IMF content in growing-finishing pigs [9]. Once generated, approximately 60%–70% of SCFAs are directly absorbed by colonic epithelial cells for energy supply [52], while the remainder enters the portal circulation and, after hepatic metabolism, acts on multiple peripheral organs including skeletal muscle and adipose tissue [53], demonstrating differential lipid metabolism regulation across tissues. In subcutaneous fat and liver, SCFAs suppress lipogenesis by downregulating key genes like *ACC*, *FASN*, sterol regulatory element-binding protein 1c (*SREBP-1c*), upregulating *CPT1* to enhance fatty acid oxidation, and activating G protein-coupled receptors (GPRs) to inhibit insulin signaling, resulting in decreased fat accumulation [54–57]. Additionally, they modulate the secretion of gut hormones glucagon-like peptide-1 (GLP-1) and Peptide YY by activating GPR41/43, suppressing appetite and promoting insulin secretion [58, 59]. In contrast, in muscle tissue, acetate and butyrate can activate the AMPK and PPAR pathways, stimulating the upregulation of peroxisome proliferator-activated receptor gamma coactivator 1-alpha (PGC-1α). This mechanism enhances mitochondrial function, thereby improving myocyte uptake and oxidation of free fatty acids [60–62], consequently regulating the dynamic balance between IMF synthesis and degradation (Fig. 2).

Epigenetic modifications represent heritable changes in gene expression without altering the DNA sequence, primarily through DNA methylation and histone modifications, which collectively alter chromatin packaging and accessibility [63, 64]. SCFAs serve as potent inhibitors of host histone deacetylases (HDACs) through direct binding and suppression of HDAC activity. This inhibition prevents the removal of acetyl groups from histone lysine residues, resulting in reduced chromatin condensation and transcriptional silencing alongside enhanced gene expression [65]. Concurrently, acetate acts as an acetyl donor for histone acetyltransferases (HATs) to promote histone acetylation [66]. Propionate and butyrate are metabolized into propionyl-CoA and butyryl-CoA, respectively, which can be catalyzed into histone lysine by HATs, establishing repressive marks that stabilize transcriptional silencing [67]. Thus, SCFAs exert dual regulatory effects on host histone modifications (Fig. 2). When functioning as an HDAC inhibitor, butyrate treatment increases histone H3 lysine 9 acetylation, upregulating transcription of glucose metabolism-related genes like glucose transporter type 4 (GLUT4), monocarboxylate transporter 1 (MCT-1) [68], consequently enhancing glucose uptake with possible secondary stimulation of fatty acid synthesis in muscle cells. SCFAs can additionally regulate DNA methylation-modifying enzymes, including DNA methyltransferases (DNMTs) and ten-eleven translocation dioxygenases, directing their action to gene promoter regions. In promoter and enhancer regions, increased DNA demethylation generally enhances transcription of certain genes. [69]. For example, dietary betaine supplementation significantly increases the abundance of *Akkermansia muciniphila*, which produce acetate and butyrate. These SCFAs inhibit DNMT activity, reducing DNA methylation at the promoter region of the host miR-378a gene. This epigenetic modification upregulates miR-378a, which subsequently targets and suppresses the pro-obesity transcription factor Yin Yang 1, ultimately reducing systemic fat deposition in mice [70]. Although studies on SCFAs-mediated epigenetic regulation of porcine IMF deposition remain limited, the existing research may suggest a potential association.

Muscle fiber type is a critical factor influencing IMF. Based on myosin heavy chain (MyHC) polymorphism, skeletal muscle fibers can be classified into four types: I, IIa, IIb, and IIx. Type I muscle fibers are rich in mitochondria and primarily rely on aerobic metabolism (slow-twitch muscles), while type II fibers depend on glycolysis (fast-twitch muscles). However, type IIa fibers, as the subtype with the strongest oxidative capacity among fast-twitch muscles, are dominated by aerobic metabolism [71]. Studies reveal that type I fibers exhibit elevated lipid metabolism genes, including *CPT2* and *PGC-1α* (involved in lipid catabolism) as well as *PLIN2*, *FABP3*, diacylglycerol o-acyltransferase1 (*DGAT1*) (participating in lipid anabolism), enabling concurrent enhancement of fatty acid oxidation and intramuscular lipid storage [72]. Therefore, muscles composed predominantly of type I fibers such as semitendinosus exhibit higher IMF content [73]. For instance, the proportion of type I fibers is significantly greater in fat-type pigs than in lean-type pigs [74]. Compared with conventional piglets, germ-free piglets exhibit a significantly lower proportion of types I and IIa, a phenomenon that may be partially attributed to reduced circulating levels of microbially derived SCFAs [75]. Han et al. [76] found that a high-fiber diet significantly increased the abundance of type I fiber-related genes in the longissimus dorsi muscle with improved meat quality (increased pH). Butyrate has been shown to promote slow-twitch fiber formation and enhance IMF deposition in finishing pigs by upregulating *PGC-1α* expression [77] (Fig. 2). These suggest that SCFAs generated under high-fiber dietary conditions may promote a shift toward fiber types associated with higher IMF deposition.

The production of SCFAs is primarily regulated by two interdependent factors: dietary fiber intake and the structural and functional composition of the host gut microbiota. Within this system, gut microbial structure, especially fiber-degrading communities such as specific Firmicutes members [78, 79], represents the more complex and challenging component to modulate. This complexity stems largely from the dominant influence of host genetic background on microbiota composition, which drives significant interbreed variations in high-fiber diet tolerance [80–82]. For instance, Jinhua pigs demonstrate remarkable roughage adaptability, primarily attributed to their gut microbiome enrichment of cellulolytic symbionts such as *Prevotella*, *Alloprevotella*, and *Ruminococcus*. Through synergistic action, these taxa effectively break down complex carbohydrates, boosting SCFAs production and aligning with host adaptation to fibrous diets [83–85]. Li et al. [86] found that alfalfa supplementation increased *Prevotella* abundance while reducing backfat thickness and improving meat quality in finishing Heigai pigs, potentially mediated by SCFAs-induced muscle fiber type transition (promoting type I rather than type IIb fibers). Similarly, in Landrace × Yorkshire pigs, nutritional stress interventions such as increased dietary neutral detergent fiber intake were shown to modulate microbial ecology (elevating *Ruminococcus*, *Akkermansia*, *Lachnoclostridium*, and *Bifidobacterium*) while concomitantly improving pork quality metrics and reducing subcutaneous adiposity, with notable alterations observed in the PPAR signaling pathway [87]. *Bifidobacterium* and *Lactobacillus* are widely recognized as dominant probiotic genera in the gut microbiota [88, 89]. Dietary interventions with β-glucan and inulin enrich these genera [90–92], consequently enhancing IMF deposition while simultaneously optimizing fatty acid composition through increased linoleic and arachidonic acid content, ultimately improving meat quality parameters including color stability and water-holding capacity [92–94]. Mechanistically, *Lactobacillus reuteri* supplementation has been demonstrated to shift longissimus dorsi fiber type composition (favoring type I over IIb fibers), while upregulating stearoyl-CoA desaturase 1 expression to enhance IMF content [95]. However, it should be noted that high-fiber diets typically have lower energy density. Thus, their impact on IMF arises from the combined effects of energy restriction and SCFA-producing bacteria.

Collectively, these findings establish that gut microbiota-derived SCFAs critically regulate IMF deposition, but their multi-tiered regulatory networks remain incompletely understood. Existing experimental models fail to replicate the physiological complexity of in vivo systems adequately. Future research needs to develop cross-scale integrated models that couple microbial metabolic dynamics, host energy allocation, and myofiber microenvironmental responses to precisely resolve the regulatory network through which SCFAs modulate IMF. Concurrently, more physiologically relevant dynamic simulation models mimicking complex in vivo interactions should be established to guide the directional optimization of IMF deposition.

### Bile acids

Bile acids play a critical role in regulating lipid metabolism by emulsifying dietary fats, which promotes their breakdown and absorption. On one hand, the gut microbiota serves as the primary mediator in determining the composition and structure of BAs. On the other hand, they in turn selectively modulate the structure and function of the gut microbiota [96]. In the intestine, conjugated BAs synthesized by the liver undergo microbiota-mediated metabolic cascades. Bile salt hydrolase (BSH) first catalyzes the deconjugation of conjugated BAs into free primary BAs, which are subsequently converted into secondary BAs by key functional enzymes such as 7α-dehydroxylase. Bacteria harboring BSH and 7α-dehydroxylase, including *Bacteroides thetaiotaomicron*, *Enterococcus faecalis*, *Lactobacillus salivarius*, and *Clostridium perfringens* serve as pivotal regulators of this metabolic cascade [97, 98] (Fig. 2). The activity of BSH profoundly influence host lipid metabolism and adiposity, yet its effects are bidirectional. For instance, *Lactobacillus* strains with high BSH activity have been shown to reduce cholesterol accumulation in vitro [99]. However, diminished enzyme activity does not invariably promote fat deposition. Zhang et al. [100] demonstrated that depleting the abundance of BSH-active bacterial genera (e.g., *Streptococcus* and *Enterococcus*) lowered total cholesterol levels in piglets. This suggests that BSH exerts dual regulatory effects on host lipid metabolism.

As critical signaling molecules, BAs modulate lipid metabolism by activating the farnesoid X receptor (FXR) and G protein-coupled bile acid receptor (TGR5) signaling pathways [101–103]. The FXR pathway, activated by primary BAs, suppresses hepatic lipogenesis and maintains bile acid homeostasis by inhibiting carbohydrate response element-binding protein and *SREBP-1c* [104–106], which are core transcription factors for hepatic lipid synthesis, driving de novo synthesis of fatty acids and triglycerides (Fig. 2). Additionally, FXR activation upregulates the secretion of fibroblast growth factor 19, which enhances hepatic PPARα and CPT1 expression, collectively reducing lipid accumulation [107]. The consequent decline in hepatic lipid synthesis may decrease the very low-density lipoprotein secretion into circulation, ultimately reducing free fatty acid delivery to muscle tissue and mitigating IMF [108]. Concurrently, TGR5 activation in the gut promotes the release of GLP-1, which enhances energy expenditure and suppresses appetite [102, 104, 109], while also modulating hepatic lipid metabolism to reduce steatosis [110]. Beyond receptor-mediated effects, bile acids directly influence adipocyte differentiation and lipid droplet storage by modulating genes like *PPARγ*. For example, cholic acid (CA) dose-dependently inhibits adipogenesis in stromal vascular fraction cells by suppressing *PPARγ* and *C/EBPα* expression [111]. Conversely, the secondary bile acid isoallolithocholic acid (IALCA) promotes lipid accumulation in 3T3-L1 adipocytes via PPARG upregulation [106]. Microbial biotransformation of bile acids structurally modulates their agonistic activity for specific receptors and target genes, ultimately exerting profound impacts on lipid metabolic outcomes.

**Fig. 3:**
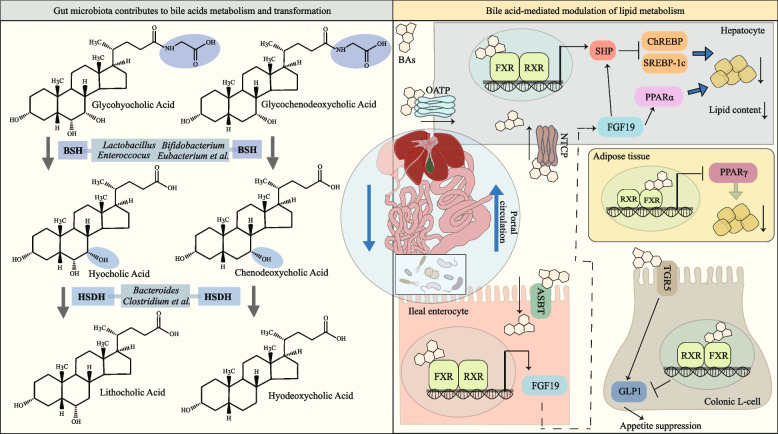
Modification of BAs by gut microbiota and their impact on host lipid metabolism. Gut microbes secrete bile enzymes BSH and HSDH, converting primary conjugated BAs into primary BAs and secondary BAs (Left). BAs secreted into the intestine can be taken up by ileal epithelial cells via the ASBT, activating FXR signaling to promote FGF19 release (Right). In the colon L-cells, BAs modulate GLP-1 secretion by activating or inhibiting TGR5 and FXR signaling, thereby regulating appetite. Free BAs and FGF19 can re-enter hepatocytes via enterohepatic circulation, promoting fatty acid oxidation while suppressing lipid synthesis. Circulating BAs further reduce lipid deposition in adipose tissue through FXR-mediated signaling. *BSH* Bile salt hydrolases, *HDSH* 7α-Dehydroxylase, *SHP* Small heterodimer partner, *ChREBP* Carbohydrate response element-binding protein, *FXR* Farnesoid x receptor, *RXR* Retinoid x receptor, *NCTP* Na⁺-taurocholate cotransporting polypeptide, *OATP* Organic anion transporting polypeptide, *FGF19* Fibroblast growth factor 19, *ASBT* Apical sodium-dependent bile acid transporter, *TGR5* G protein coupled bile acid receptor, GLP-1 Glucagon like peptide-1

Current research on the effects of BAs on lipid metabolism in pigs has primarily focused on subcutaneous and hepatic tissues. For example, Zha et al. [112] demonstrated that *Bifidobacterium pseudocatenulatum* reduces subcutaneous fat deposition in finishing pigs by increasing the abundance of secondary bile acids such as lithocholic acid, upregulating hepatic FXR and TGR5, and downregulating *PPARγ* expression in adipose tissue (Fig. 3). Hou et al. [113] found that dietary supplementation with *Lactobacillus delbrueckii* increased total BAs and upregulated *CPT1* and lipoprotein lipase (*LPL*), thereby reducing hepatic lipid content and lowering blood lipid levels. Additionally, oral administration of *Clostridium butyricum* was shown to decrease hepatic lipid accumulation by elevating taurohyocholic acid and taurochenodeoxycholic acid, activating FXR and PPARα pathways, and upregulating *CPT1* and acyl-CoA oxidase [100]. Hyodeoxycholic acid can reduce hepatic lipid accumulation and ameliorates non-alcoholic fatty liver disease by activating PPARα pathways [114, 115]. A growing body of evidence confirms the influence of bile acids and their derivatives on IMF content. Hu et al. [106] observed significant differences in bile acid profiles between fatty and lean pig breeds, with IALCA, 12-Keto-lithocholic acid, and 3-oxo-deoxycholic acid showing a strong positive correlation with IMF content. These specific bile acid molecules may affect IMF by modulating the metabolic activity of intramuscular adipocytes.

Taken together, current research has not only elucidated the multifaceted molecular mechanisms by which bile acids and their derivatives regulate lipid metabolism in subcutaneous and hepatic tissues, but emerging evidence also reveals a significant correlation between specific bile acid profiles and IMF deposition. The field could prioritize development of dynamic bile acid tracing technologies, aiming to quantify transport efficiency and metabolic fate along the gut-muscle axis. Integrating microbial enzyme profiles with bile acid libraries will enable predictive models for IMF regulation, advancing targeted strategies to optimize meat quality through precision bile acid modulation.

### Branched-chain amino acids

BCAAs, comprising leucine, isoleucine, and valine, are essential amino acids that can only be synthesized by plants and microorganisms [116]. Microbes produce BCAAs through complex metabolic pathways. Although the specific enzymes and intermediates may vary among microbial species, their biosynthetic pathways generally originate from pyruvate or acetyl-CoA, undergoing sequential reactions including transamination, dehydrogenation, and isomerization to ultimately generate these three metabolites [117]. Beyond serving as nutritional components, BCAAs actively participate in regulating host lipid metabolism [118]. Studies have identified positive correlations between BCAA levels and the abundance of specific gut microbiota, including *Bacteroides stercoris*, *Enterorhabdus, Eubacterium xylanophilum*, and *Butyricicoccus*. Inhibition of microbial BCAA synthesis has been shown to reduce host lipid deposition [119, 120]. Isoleucine promotes intracellular lipid droplet accumulation in myotubes by upregulating PPARγ and FASN while downregulating adipose triglyceride lipase and LPL protein levels [121]. During adipocyte differentiation and lipogenesis, BCAA metabolism becomes markedly enhanced. Notably, metabolites derived from leucine and isoleucine can contribute up to one-third of the acetyl-CoA supply for ACC during fat synthesis (Fig. 1) [122]. This demonstrates the crucial role of BCAA catabolism in adipocyte function and energy homeostasis, with enhanced breakdown promoting lipogenesis.

BCAAs can stimulate fatty acid transport in the blood and enhance skeletal muscle fatty acid uptake, collectively promoting lipid deposition [123]. Supplementing BCAAs in low-protein diets not only alters muscle fiber types (increasing *MyHC I* and decreasing *MyHC IIb* abundance) but also upregulates the expression of genes such as fatty acid transporter protein 1 (*FATP1*) and *PPARγ*, which promote IMF deposition in the longissimus dorsi of finishing pigs [13]. Additionally, this dietary intervention is associated with increased abundances of gut microbiota, including *Paludibacteraceae* and *Synergistaceae* [124]. Yang et al. [125] found that compared to Duroc × Landrace × Yorkshire (DLY), Ningxiang pigs harbored *Lactobacillus reuteri* as a key microbial species regulating lipid deposition, with enhanced capacity for intestinal BCAA synthesis. Oral administration of *Lactobacillus reuteri* significantly increased circulating BCAA levels in DLY pigs, and promoted IMF deposition by upregulating *SREBP-1c* expression. Chen et al. [126] revealed that *Prevotella copri* stimulates BCAA production, activating, subsequently upregulating the expression of adiponectin, *DGAT2*, *FABP3*, modulating intramuscular fat deposition.

All the above evidence demonstrates that BCAAs regulate lipid metabolism through multiple mechanisms, playing roles not only in cellular differentiation and energy metabolism but also in modulating host lipid deposition by influencing the expression of lipid metabolism-related genes. We propose that subsequent research focus on investigating the mechanisms of each BCAA individually. By integrating bacterial BCAA production capacity, host amino acid transport efficiency, and muscle metabolic response networks, this will facilitate the evolution from nutritional interventions to precision-designed microbial synthesis strategies.

### Trimethylamine N-oxide

TMAO is a small molecule compound produced mainly through two pathways: direct dietary intake which particularly rich in fish and microbial transformation of trimethylamine (TMA)-containing precursors like choline and carnitine, which are prevalent in animal products including red meat, eggs, and dairy [127, 128]. Specific gut microbes including *Lachnoclostridium* and *Clostridium*, generate TMA from these substrates via microbial lyases, with subsequent hepatic conversion to the final metabolite by flavin monooxygenase 3 following intestinal absorption [129]. Elevated circulating levels of TMAO promote systemic lipid deposition by upregulating the gene expression of ATP citrate lyase, pyruvate dehydrogenase, and *SREBP1c*, while simultaneously altering gut microbiota composition, reducing the abundance of *Bifidobacterium*, *Bacteroidetes*, and *Olsenella* [130–132]. Additionally, δ-Valerobetaine, a TMAO precursor, induces lipid accumulation through inhibition of the carnitine shuttle system, which reduces fatty acid transport into mitochondria for β-oxidation [133]. In vitro evidence from HepG2 cell models, TMAO treatment exacerbates palmitic acid-induced cellular lipid accumulation [134], collectively establishing its role in promoting adipogenesis.

Research demonstrates that dietary cholesterol significantly elevates both serum lipids and circulating levels of TMAO in finishing pigs. These changes are accompanied by increased abundances of *Bacteroides* and *Ruminococcus* along with corresponding shifts in microbial metabolic pathways favoring enhanced fatty acid and lipid biosynthesis [135]. Similarly, high-fructose high-fat diets impair choline metabolism, leading to elevated TMAO levels and subsequent hepatic steatosis in piglets [107]. Yang et al. [136] demonstrated a positive correlation between the abundance of *Proteobacteria* with elevated blood lipid and TMAO levels in Tibetan minipigs. When supplemented directly in finishing swine diets, the compound significantly augments backfat thickness and IMF deposition in longissimus dorsi through *SREBP1c* upregulation and *CPT1* downregulation, while altering fatty acid saturation profiles and enriching ileal *Proteobacteria* populations [15]. These studies indicate that dietary intake of choline and carnitine induces alterations in the gut microbiota, resulting in elevated TMAO levels.

Although it demonstrates a significant promoting effect on IMF deposition in the longissimus dorsi muscle, TMAO may concurrently lead to adipose deposition in other depots. At present, research on the regulatory mechanisms of tissue-specific TMAO deposition remains unexplored. In the future, precision nutritional intervention strategies based on microbial functional predictions could be developed, thus optimizing meat quality improvement while mitigating systemic metabolic risks associated with TMAO.

### Tryptophan related metabolites

Tryptophan is one of the twenty essential amino acids, which is an aromatic amino acid containing five double bonds and two alanine structural units. Although tryptophan is the least abundant amino acid in proteins and cells, it acts as a biosynthetic precursor for a wide range of microbial and host metabolites [137]. Gut microbiota including *Bifidobacterium bifidum*, *Clostridium sporogenes*, *Eubacterium rectale*, and *Lactobacillus paracasei* can convert tryptophan into indole and its related derivatives, such as indole-3-sulfonic acid (ISA), indole-3-acetic acid (IAA), indole-3-propionic acid (IPA), and indole-3-lactic acid (ILA) [138]. Research has demonstrated that ISA and ILA exhibit highly specific and dose-dependent inhibitory effects on adipocyte differentiation and lipid accumulation [139–141], suggesting their potential role in reducing host fat deposition. Furthermore, tryptophan metabolites can function as agonists for the aryl hydrocarbon receptor (AhR) [142], a transcription factor that regulates key gene expression in mammalian cells and plays a significant role in lipid metabolism [143]. During 3T3-L1 preadipocyte differentiation, AhR expression progressively decreases and negatively regulates adipocyte differentiation [144]. Dou et al. [145] found that AhR overexpression reduces *PPARγ* stability and inhibits adipocyte differentiation, while AhR knockout stimulates adipogenic differentiation in 3T3-L1 cells. This implies that gut microbiota may influence lipid metabolism through tryptophan metabolites that modulate the AhR signaling pathway.

Dietary supplementation with tryptophan significantly increases the abundance of IAA and related metabolites while reducing serum and hepatic TAG levels in both weaned piglets and finishing pigs, as well as decreasing backfat thickness in finishing pigs [146–148]. Geng et al. [149] found that dietary supplementation with *Lactobacillus plantarum* modulates tryptophan metabolism in weaned piglets, enhancing IAA production and lipid metabolic activity. Li et al. [141] demonstrated that time-restricted feeding promotes *Lactobacillus* colonization in growing pigs, and subsequently elevates ILA levels to activate AhR pathway while upregulating *PPARα* and *CPT1* expression, thereby reducing lipid deposition in serum and liver. Additionally, ILA produced by *Lactobacillus* can increase serum GLP-1 levels in pigs, regulating appetite and energy expenditure, possibly contributing to reduced subcutaneous and liver fat accumulation [110]. Furthermore, ILA modulates lipid metabolism through epigenetic modification of host DNA methylation. Liu et al. [111] found that dietary quercetin intervention significantly increased the relative abundance of *Akkermansia muciniphila* in the gut. Both the bacterium and its metabolite ILA reduced host m^6^A modification levels while upregulating cytochrome p450 family 8 subfamily b member 1 (CYP8B1) expression, thereby elevating serum CA abundance and attenuating lipid deposition in adipose tissue.

Therefore, microbially derived tryptophan metabolites effectively suppress fat deposition in the liver and adipose tissue. Importantly, this optimized regulation of overall lipid metabolism may improve energy partitioning to influence IMF deposition potentially. Future research should emphasize isolating and characterizing additional tryptophan-metabolizing microbial strains, and the crosstalk between tryptophan metabolism and BA/BCAA metabolic axes needs to be defined for their synergistic regulation of IMF deposition.

### Other metabolites

#### Succinate

Succinate is an intermediate or terminal metabolite produced by gut microbes like *Bacteroides*, *Prevotella*, and *Parabacteroides distasonis* (Fig. 2). Although studies in high-fat diet (HFD) mice suggest that succinate anti-obesity potential [150], other research indicates that succinate exerts anti-lipolytic effects in adipose tissue via succinate receptor 1 [151, 152]. Dietary supplementation of succinate significantly increases IMF deposition in the longissimus dorsi of finishing pigs, enhances marbling score and tenderness, and upregulates adipogenic-related genes, *FASN*, *C/EBPα*, and *PPARγ* [16]. During muscle fiber type transformation, succinate activates phospholipase Cβ, via the membrane receptor GPR91, generating the second messengers inositol 1,4,5-trisphosphate (IP₃) and diacylglycerol. This cascade elevates intracellular Ca^2^⁺ concentration, subsequently activating the Ca^2^⁺/nuclear factor of activated T-cells pathway, which promotes a shift in muscle fiber types from type IIb to type I [153].

#### Polyamine

The polyamine family, comprising putrescine, spermidine, and spermine, plays important metabolic roles. Intestinal microbial dominates like *Lactobacillus, Bifidobacterium*, *Clostridium*, and *Enterococcus* in the distal gut produce putrescine and spermidine, serving as significant polyamine sources for the host [154]. Ma et al. [155] found that *Lactobacillus reuteri* ZJ617 reduces obesity in HFD mice by enhancing microbial spermidine production. Similarly, in vitro studies reveal that spermidine and spermine inhibit *C/EBPα* expression, thereby suppressing adipocyte differentiation and lipid accumulation [156, 157]. Furthermore, spermidine elevates *PGC-1α* levels in C2C12 myotubes [17], indicating that polyamines may enhance muscle energy expenditure. However, their precise effects on IMF deposition warrant further systematic investigation.

#### Trans fatty acids

Trans fatty acids (TFAs) are a class of unsaturated fatty acids containing trans double bonds, like the conjugated linoleic acid (CLA) isomer *trans*-10, *cis*-12 (t10, c12). The gut microbiota plays a pivotal role in the metabolism of fatty acid isomers. Specific bacterial strains, such as *Bifidobacterium breve*, have been identified as capable of converting linoleic acid into CLA isomers [158]. Notably, CLA isomers also significantly modulate the composition of gut microbiota. Dietary supplementation with 1% CLA (t10, c12 isomer accounted for 40%) has been shown to increase the relative abundance of *Parabacteroides*, *Bacteroides*, and *Lachnospiraceae_UCG-010*, thus promoting lipid accumulation in the muscles of finishing pigs [46]. Similarly, Jiang et al. [159] demonstrated that 1.25%–2.5% CLA (t10, c12 isomer accounted for 37.46%) diets increased lean meat percentage and IMF content. Furthermore, Duan et al. [160] found that the t10, c12 but not c9, t11-CLA isomer enhanced oxidative skeletal muscle fiber types in mice. These findings suggest that TFAs may significantly influence porcine fat deposition and meat quality by modulating gut microbiota composition and muscle fiber type.

## Strategies for IMF modulation

### Dietary interventions

Dietary interventions effectively modulate gut microbiota to improve meat quality and enhance IMF. Beyond dietary fiber mentioned earlier, dietary protein and fatty acids also exert substantial influence. Liu et al. [161] reported that a low-protein diet with balanced amino acids elevated IMF content by enriching *Turicibacter*, *Terrisporobacter*, and *Clostridium_sensu_stricto_1*, while also altering the fatty acid and amino acid composition of the longissimus dorsi muscle. Dietary supplementation with polyunsaturated fatty acids, like CLA, has been shown to significantly increase IMF content and modify microbial community structure [159, 162]. Niu et al. [163] demonstrated that a silage diet boosted beneficial microbes, including *Clostridium_sensu_stricto_1* and *Terrisporobacter*. This microbial shift promoted muscle fatty acid synthesis and deposition, thereby enhancing IMF content and improving meat quality.

### Microbial interventions

Microbial interventions have been extensively studied to improve meat quality and promote IMF deposition. Different intestinal segments contribute to IMF through distinct mechanisms: small intestinal microbiota influence lipid digestion and absorption through phospholipases and fatty acid synthesis genes [22, 164], directly affecting IMF precursor supply. In contrast, large intestinal microbiota indirectly promotes IMF deposition by modulating host metabolism through microbial metabolites. Tang et al. [165] suggested that jejunal microbiota showed the highest microbial contribution to IMF, followed by cecal and colonic microbiota. Conversely, Jin et al. [166] identified the colon as the key intestinal segment regulating IMF deposition, with significantly higher microbial diversity and lipid metabolism pathway enrichment compared to other segments. While these intestinal segments synergistically influence IMF, their relative contributions require further investigation.

#### Fecal microbiota transplantation

In recent years, fecal microbiota transplantation (FMT) has emerged as a crucial tool for investigating the influence of gut microbiota on host physiological functions, providing key insights into microbe-host interactions. The gut microbiota of obese-type pigs exhibits a stronger capacity for fatty acid synthesis, enabling greater energy deposition and enhanced fat distribution in skeletal muscle [74]. When fecal microbiota from obese-type pigs such as Laiwu and Ningxiang pigs, were transplanted into lean-type pigs like DLY, the recipients showed a significant increase in IMF content and improved meat quality, such as higher marbling score, improved meat color, and elevated pH, along with reduced backfat thickness. This suggests that microbiota can selectively regulate fat deposition patterns [167, 168]. Yan et al. [169] found that germ-free mice receiving gut microbiota transplants from Rongchang pigs, compared to those receiving transplants from DLY pigs, exhibited higher in IMF deposition along with altered muscle fiber composition which showed increased slow and decreased fast-twitch fiber proportion.

#### Probiotic

In livestock production, microbial interventions (probiotic supplementation or fermented feed) can effectively enhance meat quality and IMF content. *Bacillus* are widely utilized probiotics. Studies demonstrate that oral administration of *Bacillus amyloliquefaciens* enhances marbling score and tenderness in finishing pigs by upregulating the expression of *LPL* and cluster of *CD36* which participate in vascular fatty acids distribution and transportation (Table 3) [178]. Similarly, *Bacillus subtilis* and *Clostridium butyricum* significantly improved meat color and marbling scores in the longissimus dorsi [170]. Feeding pigs with co-fermented feed containing *Enterococcus faecium* and *Bacillus subtilis* markedly upregulated the expression of *C/EBPα*, *PPARγ*, *SREBP1* and *FABP4* in muscle, promoting IMF deposition and increasing polyunsaturated fatty acid content, thereby modifying muscle fatty acid composition [171, 176]. These findings demonstrate that gut microbiota plays a pivotal regulatory role in skeletal muscle lipid metabolism and is indispensable in intramuscular lipogenesis.

#### Potential IMF-associated gut microbiota

In addition to gut microbial metabolites, changes in the abundance of specific microorganisms can directly influence host lipid metabolism. For instance, Xie et al. [168] found that the abundance of *Bacteroides uniformis*, *Sphaerochaeta globosa*, *Hydrogenoanaerobacterium saccharovorans*, and *Pyramidobacter piscolens* was significantly positively correlated with IMF (Table 3). These microbes promote fatty acid synthesis and storage by upregulating genes such as *FASN* and *DGAT2* [168]. *Treponema bryantii*, *Jeotgalibaca dankookensis*, and *Clostridium *sp. CAG:413 were more abundant in Laiwu pigs with higher IMF content and showed a positive correlation with fat deposition, potentially enhancing IMF deposition by regulating the phosphoinositide 3-kinase/protein kinase B signaling pathway [174, 175]. *Bacteroides plebeius* was significantly enriched in high-fat pigs, and its genome encodes glycoside hydrolase enzymes (e.g., *GH2* and *GH20*) that may participate in lipid metabolism and modulate gut cell communication, thereby influencing fat deposition [172]. In Ningxiang pigs, a higher abundance of *Lactobacillus reuteri XL0930* was observed which downregulates *SLC22A5*, reduces carnitine uptake and inhibits fatty acid β-oxidation, thereby promoting intramuscular fatty acid accumulation [167]. These findings indicate that gut microbiota significantly influences animal fat deposition, with different microbial profiles exerting distinct effects.

### Genetic interventions

While microbial and dietary interventions represent short-to-medium-term approaches for modulating gut microbiota to enhance IMF deposition, sustainable optimization requires long-term strategies incorporating host genetic regulation. Substantial evidence shows interactions between host genetics and gut microbiota, with microbial composition and specific taxa abundances being heritable traits [179, 180]. This heritability enables selective breeding programs targeting beneficial microorganisms. For instance, Tiezzi et al. [177] identified strong genetic correlations between IMF-related meat quality traits and abundances of *Prevotella* suggesting gut microbiota could serve as cost-effective indicator traits for genetic improvement of pork quality. Advanced approaches like microbial genome-wide association studies and metagenomic association analyses can identify host genetic loci significantly associated with specific microbial taxa abundances [10, 181]. These loci may serve as molecular markers for long-term breeding selection. In conclusion, systematically optimizing IMF deposition requires integrating microbial, dietary and genetic microbiota modulation strategies.

## Limitations of current research and future perspectives

In modern meat production, consumer demand for meat quality is continuously increasing. There is an urgent need to promote IMF deposition to obtain meat products with improved flavor. Although significant progress has been made in studying the association between animal intramuscular fat and gut microbiota (Tables 2 and 3), this field still has many shortcomings. Firstly, the colonization pattern of the host microbiota during early life may have a profound impact on IMF deposition in adulthood through epigenetic modifications or metabolic programming. However, long-term tracking studies on early microbiota interventions are extremely scarce. Most existing studies are based on short-term intervention experiments that range from weeks to months, making it challenging to analyze the sustained effects of microbial community dynamics on fat deposition. For example, short-term supplementation of *Lactobacillus reuteri* can temporarily alter the microbiota structure and affect IMF content [164], but its long-term effects remain unclear after the microbiota returns to homeostasis. Secondly, although some current studies have utilized multi-omics technologies like genomics, metagenomics, and metabolomics to explore targets for IMF deposition and the regulatory mechanisms of microbiota on host intramuscular fat deposition [182–184], they have not validated the direct regulatory mechanisms through in vitro experiments. Further research could potentially combine gene editing, muscle-organoid co-culture, and metabolomics-proteomics integration to systematically dissect the causal regulatory network of "microbiota-metabolite-host target".

**Table 2:** Microbial metabolites mediate host fat deposition

Metabolites	Up/Down fat deposition	Mechanism	Other phenotypes	Methods	Citation
SCFAs	↓Subcutaneous fat; ↑IMF	Activate GPR41, GPR43 and modulate the secretion of GLP-1 and PYY	Increase meat quality and promote muscle fiber type transformation	Multi-omics; Molecular biology techniques	[77, 87,95,86 ]
CA	↓Liver fat	Activate the FXR signaling pathway and suppresses *ChREBP*, *SREBP-1c*,* PPARγ*	—	Multi-omics; Molecular biology techniques	[104, 106, 111]
LCA	↓Subcutaneous fat	Suppress *PPARγ*	—	Multi-omics; Molecular biology techniques	[112]
HDCA	↓Liver fat	Activate PPARα	—	Correlation analysis	[114, 115]
CDCA	↓Liver fat	Activate the FXR/PPARα signaling pathway	—	Multi-omics; Molecular biology techniques	[100]
IALCA	↑IMF	Upregulate *PPARγ*	—	Molecular biology techniques; Correlation analysis	[106]
3-oxo-DCA	↑IMF	—	—	Correlation analysis	[106]
12-KLCA	↑IMF	—	—	Correlation analysis	[106]
Leu	↑IMF	Increase *FATP1*, *PPARγ, SREBP-1c*	Transfer fiber characteristics	Multi-omics; Molecular biology techniques	[13, 125]
Ile	↑IMF	Increase *FATP1*, *SREBP-1c,* PPARγ*;* reduce ATGL, LPL	Transfer fiber characteristics	Multi-omics; Molecular biology techniques	[13, 121, 125]
Val	↑IMF	Increase *ADIPOQ*, *DGAT2*; Increase *FATP1*, *PPARγ, SREBP-1c*	Transfer fiber characteristics	Multi-omics; Molecular biology techniques	[13, 125]
ILA	↓Liver fat	Activate the AhR and upregulate *PPARα* and *CPT1*	—	Multi-omics; Molecular biology techniques	[141]
IAA	↓Liver fat	—	—	Correlation analysis; Molecular biology techniques	[147, 149]
TMAO	↑Subcutaneous fat; ↑IMF	Increase *ACL*, *PDH*, *SREBP1c*; reduce the β-oxidation	Alter the ratio of fatty acids to unsaturated fatty acids in meat	Multi-omics; Correlation analysis; Molecular biology techniques	[15, 132, 133]
Succinate	↑IMF	Increase *FAS*, *C/EBPα*, *PPARγ*	Transfer fiber characteristics	Molecular biology techniques	[16, 153]
TFAs	↑IMF	—	Improve meat color scores, pH	Multi-omics; Correlation analysis	[46]

*SCFAs* Short-chain fatty acids, *CA* Cholic acid, *LCA* Lithocholic acid, *HDCA* Hyodeoxycholic acid, *CDCA* Chenodeoxycholic acid, *IALCA* Isoallolithocholic acid, *3-oxo-DCA* 3-oxo-deoxycholic acid, *12-KLCA* 12-keto-lithocholic acid, *ILA* Indole-3-propionic acid, *IAA* Indole-3-acetic acid, *TMAO* Trimethylamine N-oxide, *GPR41/43* G protein-coupled receptor 41/43, *FXR* Farnesoid X receptor, *TGR5* Takeda G protein-coupled receptor 5, *GLP-1* Glucagon-like peptide-1, *FGF19* Fibroblast growth factor 19, Other abbreviations as in Table 1

**Table 3:** Microbiota mediated host fat deposition

Gut microbiota	Up/Down fat deposition	Mechanism	Other phenotypes	Methods	Citation
*Akkermansia*	↓Subcutaneous fat	Produce SCFAs	Improve meat color scores, pH and tenderness	Correlation analysis	[87]
*Bacillus amyloliquefaciens*	↑IMF	Upregulate *LPL*, *CD36*	Transfer fiber characteristics	Molecular biology techniques	[46]
*Bacillus subtilis*	↑IMF	Increase *C/EBPα*, *PPARγ*, *SREBP1*, *FABP4*	Increase meat color and marbling score; Improve muscle fatty acid composition	Molecular biology techniques	[170, 171]
*Bacteroides plebeius*	↑IMF	—	—	Correlation analysis	[172]
*Bacteroides uniformis*	↑IMF	Upregulate *FASN*, *DGAT2*	—	Multi-omics; Correlation analysis	[168]
*Bifidobacterium*	↓Subcutaneous fat; ↑IMF	Produce SCFAs	Improve meat color scores, drop loss and the ratio of saturated to unsaturated fatty acids	Multi-omics; Molecular biology techniques; Correlation analysis	[87, 92, 93]
*Bifidobacterium pseudocatenulatum*	↓Subcutaneous fat	Increase LCA	—	Multi-omics; Molecular biology techniques	[112]
*Clostridium butyricum*	↓Subcutaneous fat; ↑IMF	Produce SCFAs; Increase THCA and TCDCA	Increase ribeye area; Increase meat color and marbling score	Microbiota Intervention; Molecular biology techniques	[100, 170, 173]
*Clostridium_sensu_stricto_1*	↑IMF	—	Improve drop loss and tenderness	Correlation analysis	[161, 163]
*Clostridium *sp. CAG:413	↑IMF	Activate PI3K/AKT signaling pathway	—	Multi-omics; Correlation analysis	[174, 175]
*Enterococcus faecium*	↑IMF	Increase *C/EBPα, PPARγ, SREBP1, FABP4*	Improve muscle fatty acid composition	Molecular biology techniques	[171, 176]
*Hydrogenoanaerobacterium saccharovorans*	↑IMF	Upregulate *FASN*, *DGAT2*	—	Multi-omics; Correlation analysis	[168]
*Jeotgalibaca dankookensis*	↑IMF	Activate PI3K/AKT signaling pathway	—	Multi-omics; Correlation analysis	[74, 175]
*Lachnospiraceae_UCG-010*	↑IMF	—	—	Correlation analysis	[46]
*Lactobacillus*	↓Subcutaneous and liver fat; ↑IMF	Produce SCFAs; Increase ILA	Improve the ratio of fatty acids to unsaturated fatty acids in meat	Multi-omics; Molecular biology techniques; Correlation analysis	[90, 141]
*Lactobacillus reuteri*	↑IMF	Increase BCAAs; Upregulate *SCD*	Transfer fiber characteristics	Multi-omics; Molecular biology techniques;	[95, 125, 167]
*Lachnoclostridium*	↓Subcutaneous fat	Produce SCFAs	Improve meat color scores, pH and tenderness	Multi-omics; Correlation analysis	[87]
*Megasphaera*	↓Subcutaneous fat; ↑IMF	Produce SCFAs	Improve meat color scores, drop loss	Multi-omics; Correlation analysis	[91, 92, 94]
*Parabacteroides*	↑IMF	—	—	Correlation analysis	[46]
*Prevotella*	↑IMF	Produce SCFAs	Improve marbling score	Statistics; Multi-omics; Correlation analysis	[9, 177]
*Prevotella copri*	↑IMF	Produce SCFAs, BCAAs	—	Multi-omics; Correlation analysis	[126]
*Pyramidobacter piscolens*	↑IMF	Upregulate *FASN*, *DGAT2*	—	Multi-omics; Correlation analysis	[168]
*Ruminococcus*	↓Subcutaneous fat; ↑IMF	Produce SCFAs; Increase *ACACA*, decrease *CPT1*	Improve meat color scores, pH and tenderness; Transfer fiber characteristics	Multi-omics; Correlation analysis	[87, 169]
*Sphaerochaeta globosa*	↑IMF	Upregulate *FASN*, *DGAT2*	—	Multi-omics; Correlation analysis	[168]
*Terrisporobacter*	↑IMF	—	Improve drop loss and tenderness	Correlation analysis	[161, 163]
*Treponema bryantii*	↑IMF	Activate PI3K/AKT signaling pathway	—	Multi-omics; Correlation analysis	[174, 175]
*Turicibacter*	↑IMF	—	Improve drop loss and tenderness	Correlation analysi	[161]

Abbreviations as in Tables 1 and 2

Current challenges in gut microbiota-mediated IMF regulation lie in the instability of microbial colonization and the transient efficacy of metabolite interventions, hindering sustained and stable modulation. In future production, mucosa-adhesion enhancing prebiotics can be leveraged to improve colonization capacity of key bacteria, while sustained-release microcapsule systems may prolong the action duration of metabolites for long-term effects. Additionally, engineered probiotics overexpressing IMF-related metabolic enzymes and secreting specific signaling molecules could directly target muscle tissue. Ultimately, host genomic factors like genetic background, polymorphisms, and expression, directly or indirectly regulate gut microbiota composition [177, 179, 180]. Integrating microbiome-assisted breeding to select **‘**high IMF-microbiota synergy**’** genotypes will enable stable, long-term microbial modulation through host-microbe trait coupling. Nevertheless, critical hurdles including microbial functional redundancy, immune tolerance risks from prolonged interventions, and host-microbe cross-tissue crosstalk mechanisms require deeper investigation. In summary, research on intramuscular fat-gut microbiota interactions is transitioning into a new era of mechanistic dissection and precision regulation, advancing sustainable animal husbandry.

## Conclusion

Flavorful meat products have always been a goal in livestock production, and intramuscular fat is a vital indicator affecting meat flavor and quality. This review summarizes the impact of gut microbiota and their metabolites on body fat deposition and intramuscular fat deposition in animals. It provides new insights and theoretical foundations for improving meat quality by regulating gut microbiota, offering potential strategies for optimizing meat quality in livestock production.

## Data Availability

No data was used for the research described in the article.

## Abbreviations

ACC: Acetyl-CoA carboxylase
AhR: Aryl hydrocarbon receptor
AMPK: AMP-activated protein kinase
BAs: Bile acids
BCAAs: Branched-chain amino acids
BSH: Bile salt hydrolases
CA: Cholic acid
C/EBPs: CCAAT/enhancer-binding proteins
CD36: Cluster of differentiation 36
CDCA: Chenodeoxycholic acid
CIDEC: Cell death-inducing DFFA-like effector c
CPT1: Carnitine palmitoyltransferase 1
CYP8B1: Cytochrome P450 family 8 subfamily B member 1
DGAT: Diacylglycerol O-acyltransferase
DLY: Duroc × Landrace × Yorkshire
DNMT: DNA methyltransferase
FABP: Fatty acid-binding protein
FASN: Fatty acid synthase
FATP1: Fatty acid transport protein 1
FGF19: Fibroblast growth factor 19
FMT: Fecal microbiota transplantation
FXR: Farnesoid X receptor
GH: Glycoside hydrolase
GLP-1: Glucagon-like peptide-1
GLUT4: Glucose transporter type 4
GPRs: G protein-coupled receptors
HATs: Histone acetyltransferases
HDACs: Histone deacetylases
HFD: High-fat diet
IALCA: Isoallolithocholic acid
IAA: Indole-3-acetic acid
ILA: Indole-3-lactic acid
IMF: Intramuscular fat
IP3: Inositol 1,4,5-trisphosphate
LCA: Lithocholic acid
LPL: Lipoprotein lipase
MCT1: Monocarboxylate transporter 1
MyHC: Myosin heavy chain
PGC-1α: Peroxisome proliferator-activated receptor gamma coactivator 1-alpha
PLIN: Perilipin
PPAR: Peroxisome proliferator-activated receptor
SCFAs: Short-chain fatty acids
SREBP-1c: Sterol regulatory element-binding protein 1c
TAG: Triacylglycerol
TFAs: Trans fatty acids
TGR5: G protein-coupled bile acid receptor
TMA: Trimethylamine
TMAO: Trimethylamine N-oxide
